# Three-Dimensional Quantitative Intracellular Visualization of Graphene Oxide Nanoparticles by Tomographic Flow Cytometry

**DOI:** 10.1021/acs.nanolett.1c00868

**Published:** 2021-07-07

**Authors:** Daniele Pirone, Martina Mugnano, Pasquale Memmolo, Francesco Merola, Giuseppe Cesare Lama, Rachele Castaldo, Lisa Miccio, Vittorio Bianco, Simonetta Grilli, Pietro Ferraro

**Affiliations:** † Institute of Applied Sciences and Intelligent Systems “E. Caianiello”, CNR-ISASI, Via Campi Flegrei 34, 80078 Pozzuoli, Napoli, Italy; ‡ Department of Electrical Engineering and Information Technologies (DIETI), University of Naples “Federico II”, via Claudio 21, 80125 Napoli, Italy; § Institute of Polymers, Composites and Biomaterials, CNR-IPCB, Via Campi Flegrei 34, 80078 Pozzuoli, Napoli, Italy

**Keywords:** nanographene oxide, cellular uptake, tomographic flow cytometry, 3D intracellular spatial distribution of nanoparticles, single cell 3D microscopy

## Abstract

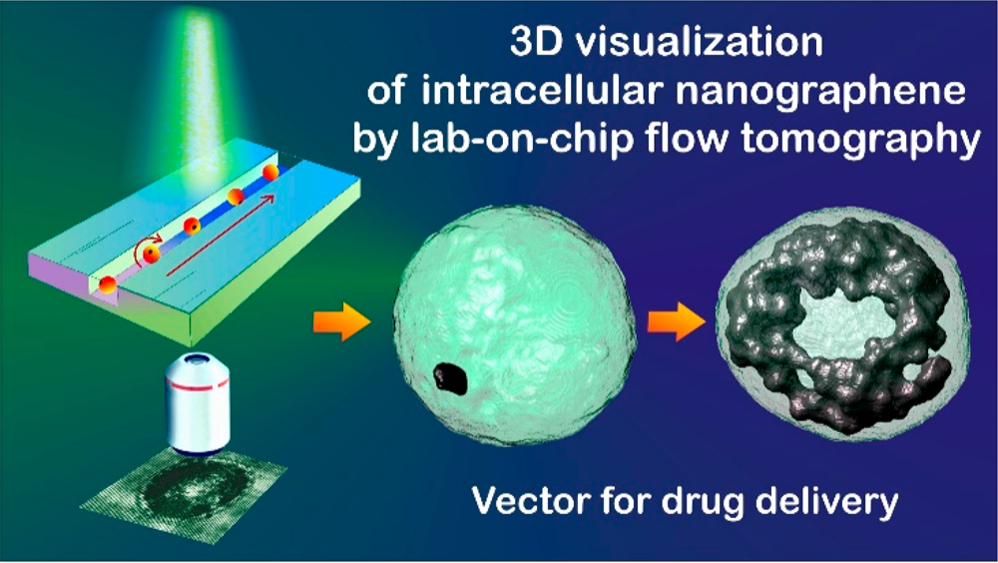

Interaction of nanoparticles (NPs) with cells is of fundamental importance in biology and biomedical sciences. NPs can be taken up by cells, thus interacting with their intracellular elements, modifying the life cycle pathways, and possibly inducing death. Therefore, there is a great interest in understanding and visualizing the process of cellular uptake itself or even secondary effects, for example, toxicity. Nowadays, no method is reported yet in which 3D imaging of NPs distribution can be achieved for suspended cells in flow-cytometry. Here we show that, by means of label-free tomographic flow-cytometry, it is possible to obtain full 3D quantitative spatial distribution of nanographene oxide (nGO) inside each single flowing cell. This can allow the setting of a class of biomarkers that characterize the 3D spatial intracellular deployment of nGO or other NPs clusters, thus opening the route for quantitative descriptions to discover new insights in the realm of NP–cell interactions.

In the last few decades, nanographene and its derivatives have captured much attention due to their superb electronic properties^1,2^ and promising applications in biomedicine field, even including approaches to fight or detect infections caused by the new coronavirus SARS-CoV-2 (COVID-19).^3,4^ Indeed, graphene and graphene oxide (GO) have been used in making DNA-based optical sensors for drug delivery^5^ and for the detection of nucleic acids,^6^ proteins,^7^ virus,^8^ metal ions,^9^ and small molecules.^10^ Furthermore, nGO is a promising candidate as vaccine carrier and adjuvant for efficient intracellular vaccine protein delivery.^11−13^ Different techniques are continuously developed and refined to study the interactions of Graphene Family Materials (GFMs) with biological samples. The MTT (3-(4,5-dimethylthiazol-2-yl)-2,5-diphenyltetrazolium bromide) cell proliferation assay is used for the nonradioactive and spectrophotometric quantification of the cell proliferation, the viability in cell populations, and the in vitro toxicology.^14^ A careful validation of MTT assay procedures is needed in experiments where GFMs are one of the constituents, to avoid a potential bias in concluding results of cytotoxicity studies.^15^ In fact, GO is a universal fluorescence quencher.^11,16^ Hence, the use of fluorescence techniques for revealing, quantifying, and visualizing GO can be affected by the fluorescent quenching due to the interactions of NPs with fluorophores and organic dyes.^17^ The gold standard technique to study nanomaterials–cells interaction or even mapping the GO intracellular distribution exploits electron-based microscopy, such as transmission electron microscopy (TEM). Furthermore, many interesting developments have been achieved recently about other imaging modalities for measuring intracellular processes^18,19^ as in confocal microscopy,^20^ multimodal optical-electron imaging,^21^ and hybrid Raman fluorescence spectral imaging method.^22,23^ However, despite all the above-mentioned methods providing imaging and measurements in cells, very few studies were devoted to visualize the 3D spatial distribution of NP uptake in a quantitative way. Confocal microscopy would be the elective optical tool to this aim. Indeed, it has been demonstrated that confocal Raman imaging can be used for tracking the nGO cellular uptake in living cells avoiding any additional fluorescent or plasmonic tag.^24^ However, while it is quite easy to perform 3D confocal scanning of cells on a flat surface, it is impossible on suspended or flowing cells. Flow cytometry measures the scattering and fluorescence signals of single cells in flow. Sample preparation is fast and simple, and statistically relevant results can be obtained because of its high-throughput property. Flow-cytometry has been demonstrated being a suitable technology to provide quantitative measurements of the cellular uptake of NPs,^25−28^ because the side scattering has been correlated to the cellular granularity.^25,26^ Unfortunately, such technology does not allow the retrieval of the exact localization of NPs into the cell volume. Alternatively, the fluorescence signal of labeled NPs provides a more robust analysis,^26,27^ but fluorescent tags can influence the particle properties and behavior. Digital holography (DH) has been recently adopted as a valuable full-field, label-free, noninvasive and high-resolution tool for nanomaterial toxicity and cell interaction studies by morphologic characterization.^29,30^ Biophysical and morphological parameters such as cell volume, thickness, density, dry mass, refractive index (RI) variation in time, and biodistribution of NPs inside cell cytoplasm can be measured by phase-contrast images without the use of chemical compounds that could interfere with nanomaterials. The ability of DH to evaluate the biodistribution of nGO internalized in adhered live cells for 24 and 48 h has been demonstrated.^31^ However, there is still a strong lack of understanding of the true 3D spatial distribution within adherent cells.^31^ In the previous studies, analysis was limited to 2D spatial distribution of nGO.^29^ On the other side, very interesting results have been presented in the DH framework by introducing a genetically encodable phase-contrast agent based on gas vesicles to enhance the contrast inside cells.^32^ To achieve a full 3D visualization of flowing cells, tomographic flow cytometry (TFC) by DH has been demonstrated to measure the morphological parameters of red blood cells, marine algae (i.e., diatoms),^33^ and circulating tumor cells.^34^ Nevertheless, nGO and their aggregates strongly absorb visible light, therefore phase-contrast microscopy cannot be effective in retrieving their 3D spatial distribution. To overcome this issue, here we propose an alternative strategy to perform in-flow cyto-tomography by DH for revealing the 3D spatial intracellular distribution of nGO. This is based on the ability of DH to automatically retrieve 3D positions and viewing angles of flowing and rotating cells along a microfluidic channel. We demonstrate that a 3D tomogram can be obtained by using the amplitude maps computed from the recorded digital holograms, thus furnishing a complete visualization in 3D of the internalized nGO aggregates. In particular, we show how the 3D spatial distribution of nGO changes in different fibroblast cells for two cell culture times, that is, 24 and 48 h. Moreover, we compare qualitatively our results with a well-established method to estimate the 3D shape of a sample, namely shape from silhouette (SFS).^35^ We show that the proposed technique allows for a much more detailed reconstruction of the 3D nGO distribution if compared to SFS. Finally, we propose some geometrical descriptors to characterize the 3D intracellular spatial distributions of these NPs. Our results open the route to high-throughput investigations at single-cell level thanks to the proofed ability to operate with flow-cytometry. Thus, it will be possible to provide statistically relevant investigations on a large number of cells for understanding how internalized nGO distributes inside each cell.

Murine embryonic fibroblasts NIH-3T3 were chosen to analyze the effects of nGO *in vitro*. The cell culture was monitored at 24 and 48 h, as shown by the inverted microscope images in Figure 1a and Figure S1a,b. At time points of 24 and 48 h, cells were detached by trypsin-EDTA and injected into a microfluidic channel to collect images of flowing cells through a DH microscope, sketched in Figure 1b. More details about the cell culture and the imaging setup are reported in the Supporting Information. In Figure 1c, we show five cuts taken from the recorded holographic sequence of a flowing and rotating NIH-3T3 cell after a treatment of 24 h with nGO. The holographic reconstruction processing based on the angular spectrum method is employed to obtain the in-focus complex wavefront, from which the amplitude map (AM) and the quantitative phase map (QPM) are recovered.^36^ For each flowing and rotating cell, the phase contrast TFC (PC-TFC) reconstruction can be performed by calculating both their 3D positions and orientations. In particular, 3D positions within the microchannel are retrieved through a holographic tracking algorithm.^37^ Each ROI containing the analyzed cell in the in-focus QPM sequence is realigned in the corresponding transversal position to avoid tomographic motion artifacts. Then, the unknown viewing angles, that correspond to the cell rolling angles, are estimated by using the computed 3D positions and by exploiting the image similarity through local contrast image measurements.^38^ Finally, the realigned images and the retrieved orientations are used as inputs of the filtered back projection (FBP) algorithm^39^ to reconstruct the 3D RI tomogram of the analyzed cell. More details about the TFC processing pipeline are reported in Supporting Information.

**Figure 1:**
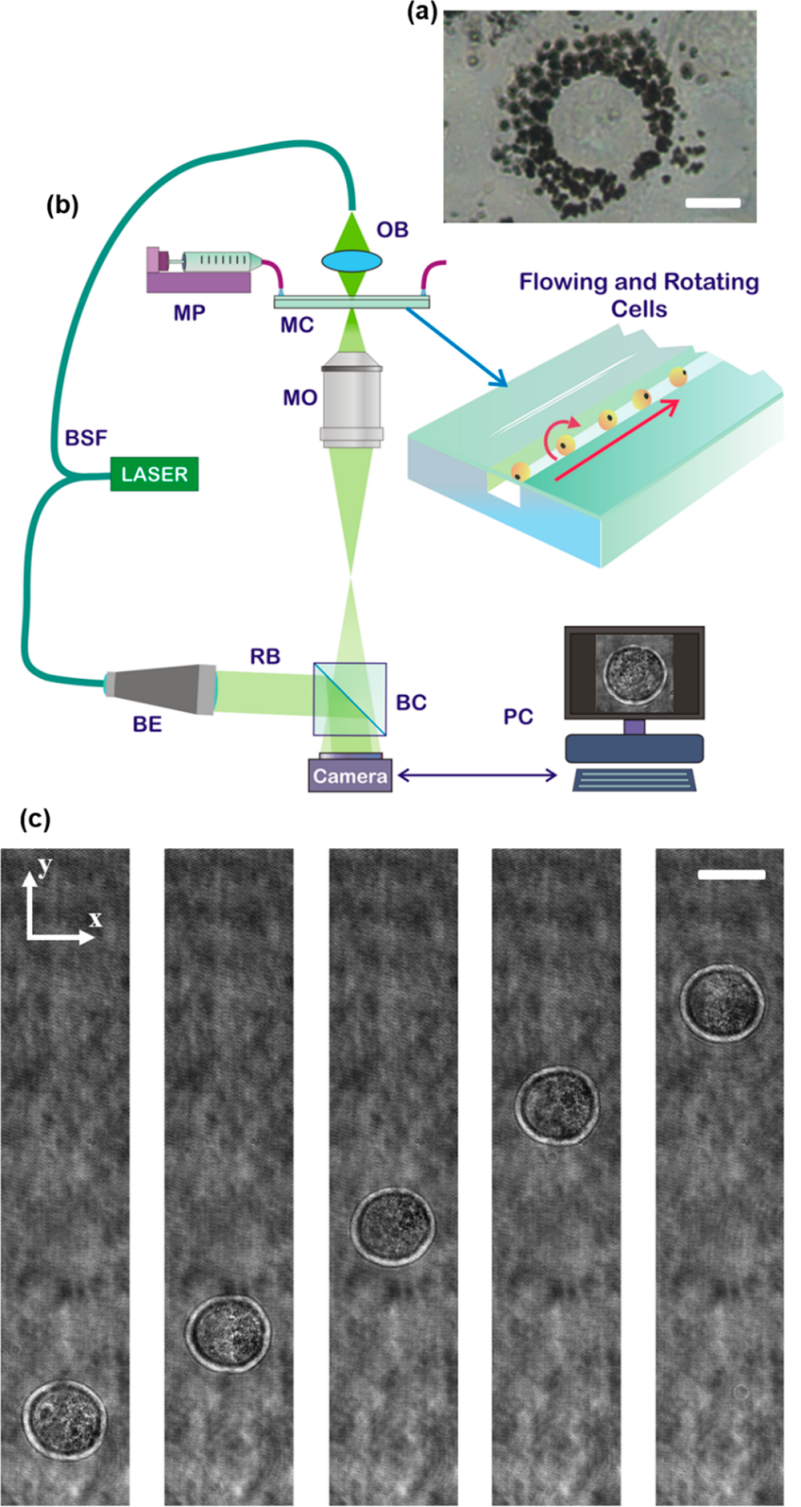
(See Supporting Movie 1) Two-dimensional imaging of NIH-3T3 cells. (a) Inverted microscope image of an NIH-3T3 cell after 48 h from the nGO adding in DMEM medium. The internalized nGO (black) distributes around the nucleus. Scale bar is 10 μm. (b) Sketch of the opto-fluidic recording system based on a DH microscope in off-axis configuration. BSF, beam splitter fiber; MP, microfluidic pump; MC, microfluidic channel; OB, object beam; MO, microscope objective; BE, beam expander; RB, reference beam; BC, beam combiner; PC, personal computer. (c) Five cuts of digital holograms of the same NIH-3T3 cell at different time frames after 24 h from the nGO adding in DMEM medium. Cell flows along the *y*-axis and rotates around the *x*-axis. Scale bar is 20 μm.

Unlike cells, nGO can is an absorbing material in the visible spectrum, which causes alterations within the periodic interference fringe pattern recorded by DH. When the amount of internalized graphene is low, this disruptive phenomenon is localized in a little region within the cell, as highlighted in Figure 2a,f,k by the blue insets within the digital holograms of three NIH-3T3 cells treated with nGO for 24 h. The effect of nGO can be observed as a dark spot in the red insets within the retrieved QPMs shown in Figures 2b,g,l. Moreover, this dark spot appears in several images of the QPM sequence, changing its position every time because of the cell rotation (see also Supporting Movies 2 and 3). After performing the PC-TFC reconstruction on the first two cells in Figure 2, the 3D visualization of accumulated nGO is obtained by setting a suitable threshold that allows its recognition at the lowest RI values, as shown in Figure 2c,h by black regions within the red cell shells (see also Supporting Movies 2 and 3). Instead, a third NIH-3T3 cell recorded with the same experimental conditions (i.e., after 24 h from the nGO adding in the DMEM medium) is reported in Figure 2k–o, where the high light absorption caused by the nGO accumulation led to a greater loss of information among all of the holograms of the recorded sequence. Indeed, the effect of light absorption in some sample areas causes the loss fringes in the hologram, as shown in the blue inset in Figure 2k. As detailed in the following, this loss of information in digital holograms provokes distortions in most of the corresponding QPMs, thus making the visualization of nGO grains unfeasible in the PC-TFC reconstruction (see also Supporting Movie 4). In fact, in Figure 2m the PC-TFC reconstruction lacks the nGO cluster, even if the graphene internalization is clearly visible in some QPMs of the sequence, as displayed in Figure 2l. To overcome this drawback, we propose to adopt AMs instead of QPMs to reconstruct the 3D tomogram, because they can collect the light absorption information much better. Figure 2d,i,n shows the AMs for the three analyzed test cases in which the nGO grains are still visible in any image of the sequence but without the abrupt jumps that characterize the corresponding QPMs (see also Supporting Movie 4). We implement the FBP algorithm with the AMs for the same angles calculated by using the rolling angles recovery method explored for PC-TFC. Therefore, we can refer to the proposed method as AM-TFC. While in PC-TFC the reconstructed tomogram is the quantitative 3D spatial distribution of cell RI, in AM-TFC we can only claim that the reconstructed tomogram is a 3D visualization of intracellular regions having different light attenuation coefficients. However, the lack of artifacts in the AMs allows the complete identification of the 3D graphene spatial distribution within the tested NIH-3T3 cells by means of AM-TFC also in the third analyzed cell for which PC-TFC fails, as reported in Figure 2o. Moreover, as proof of the effectiveness of the proposed approach, the AM-TFC reconstructions about the other two test cases in Figure 2e,j show remarkable similarities with the PC-TFC ones in Figure 2c,h, respectively, but at the same time it is clear that AM-TFC improves the 3D visualization of the nGO cluster, which instead is incomplete in PC-TFC because of the loss of information in the generative QPMs. Here, it is worth pointing out that the higher the quantity of internalized nGO, the less the ability to provide an effective PC-TFC reconstruction. Therefore, in the case of huge nGO internalization, the proposed AM-TFC reconstruction method is the key-approach for detecting accurately the 3D spatial distribution of nGO within cells. To prove this, we also study a NIH-3T3 cell after a 48 h treatment in which a massive nGO internalization can be observed. This is evident in the four frames taken from the recorded holographic sequence displayed in Figure 3a. Graphene has arranged as a ring within the cell around the nucleus (nuclear decoration^27,31^), thus occupying a large cell volume. Consequently, phase retrieval fails for any hologram of the sequence, thus preventing the PC-TFC reconstruction, as displayed in Figure 3b.

**Figure 2:**
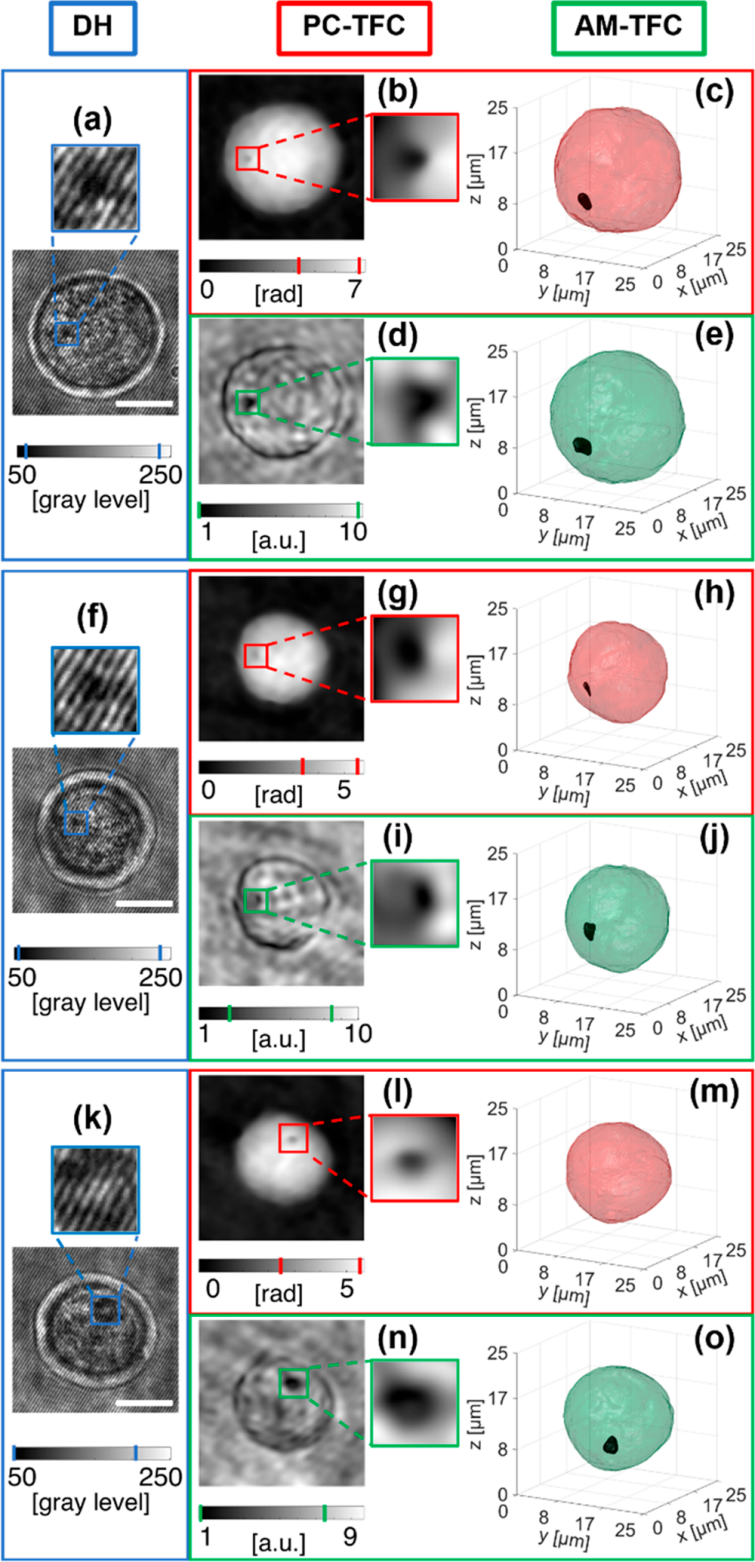
(See Supporting Movies 2, 3, and 4) Three-dimensional graphene reconstruction through PC-TFC and AM-TFC methods in three NIH-3T3 cells after 24 h treatment with nGO. (a,f,k) ROIs containing the NIH-3T3 cells in the recorded digital holograms in which the accumulation of internalized nGO has a disruptive effect on the interference fringe pattern (blue insert). (b,g,l) Numerically retrieved QPMs with highlights in red (inset), the dark spot due to graphene disturbance in the recorded holograms in (a,f,k). (c,h,m) Isolevel representation of the 3D PC-TFC reconstructions. In (c,h), the nGO accumulation (black) is segmented and isolated from the outer cell (red), while this is not possible in (m). (d,i,n) Numerically retrieved AMs, with highlights in green (inset), the dark spot due to graphene disturbance in the recorded holograms in (a,f,k). (e,j,o) Isolevel representation of the 3D AM-TFC reconstructions in which the nGO accumulation (black) is segmented and isolated from the outer cell (green). Scale bars are 10 μm. The intervals highlighted in the colorbars refer to the insets.

**Figure 3:**
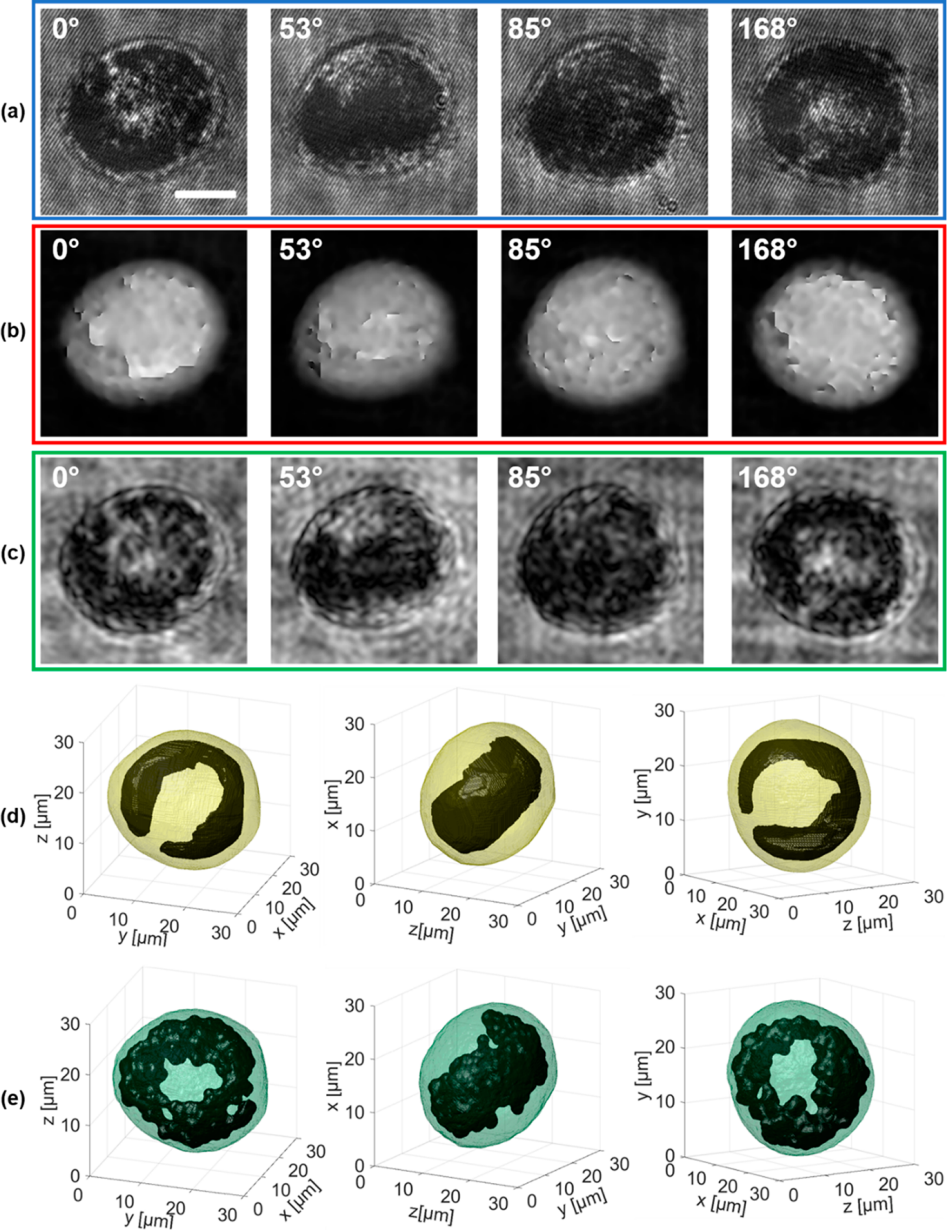
(Supporting Movie 5) Three-dimensional graphene reconstruction in an NIH-3T3 cell after 48 h treatment with nGO. (a) Four frames taken from the recorded holographic sequence of the rolling cell, from which a ring-shaped nGO spatial distribution can be inferred. (b) QPMs numerically retrieved from (a), in which the phase jumps are not corrected by the unwrapping algorithm. (c) AMs numerically retrieved from (a), in which the dark regions are due to the light attenuation of the internalized graphene. (d,e) Three views of the isolevels representation of the tomogram reconstructed through SFS and AM-TFC algorithms, respectively, in which the internalized nGO (black), segmented and isolated from the outer cell (yellow and green, respectively), distributes as a 3D ring, as observed in 2D images in (a–c). Scale bar in (a) is 10 μm. In (a–c), the estimated viewing angles are reported at the top.

In fact, when the cluster absorbs too much light, the signal collected by the sensor is too low to properly detect the object wavefront modulation of the fringe’s carrier. As a result, information in that area is lost and in turn the phase-contrast map signal cannot be retrieved. The phase signal is discontinuous in this sense, since it varies from defined values to undefined values. If phase unwrapping is performed to obtain the quantitative optical thickness map, this will show unreliable values in that area and the unwrapping error might propagate also in different areas.^40^ Thus, one cannot rely on the phase-contrast map in the presence of highly absorbing clusters for achieving quantitative information. Instead, the dark ring region is properly preserved in the AMs in Figure 3c, thus the AM-TFC approach is able to recover the 3D visualization of internalized nGO with high accuracy, as shown by three different views of the AM-TFC reconstruction in Figure 3e. To further demonstrate the effectiveness of AM-TFC algorithm, we perform a comparison with a well-established 3D shape reconstruction method, namely SFS, already demonstrated to recover the 3D visualization of live cell.^41^ Here, the SFS algorithm is performed separately on the overall cell, to reconstruct the cell shell, and on the nGO distribution obtained from AMs. The result, reported in Figure 3d, clearly shows low resolution in defining the nGO shape if compared to the AM-TFC reconstruction in Figure 3e.

The tomographic results reported in Figure 3 allow a much more complete understanding of the nGO internalization process in respect to the previous 2D methods.^31^ In fact, by means of the approach presented here, a 3D visual analysis is immediately available, thus furnishing insight on how nGO is clustering and spatially distributing within the cell volume. Besides, beyond this very useful full 3D direct visualization, here we discuss how to extract quantitative 3D measurements for a full nGO characterization. These quantitative parameters can be the basis for the definition of biomarkers for nGO-cell interaction in terms of 3D spatial intracellular deployment of nGO. In the aim to define some 3D morphological parameters to investigate nGO positioning and shapes, we report again in Figure 4a–c the AM-TFC reconstructions at 24 h of Figure 2e,j,o and we name them cell 1, cell 2, and cell 3, respectively. In particular, we measure the graphene–cell normalized distance δ(*G*,*C*), the graphene–membrane normalized distance δ(*G*,*M*), the orientation of the detected nGO cluster (i.e., the graphene–cell angle θ_GC_), its sphericity^42^ Ψ_G_, and its equivalent radius ρ_G_. In all three cases, nGO clusters are about in the same relative position between cell centroid and cell membrane, as shown by the δ(*G*,*C*) versus δ(*G*,*M*) plot in Figure 4d, and moreover they are closer to the cell membrane, as displayed in Figure 4a–c. Their sphericity Ψ_G_ decreases with a bigger graphene equivalent radius ρ_G_, as reported in Figure 4e, and with a bigger graphene-cell angle θ_GC_, as shown by polar plot in Figure 4f. In addition, as also visible in Figure 4a–c, nGO clusters are oriented about orthogonally with respect to the cell radius, but graphene–cell angle θ_GC_ slightly decreases with the graphene–cell normalized distance δ(*G*,*C*), as reported in the polar plot in Figure 4g. Hence, passing from 24 to 48 h, as the volumes of nGO clusters increase, their sphericities reduce since they stretch orthogonally with respect to cell radius, in order to finally form a unique 3D ring structure, as visible in Figure 3e.

**Figure 4:**
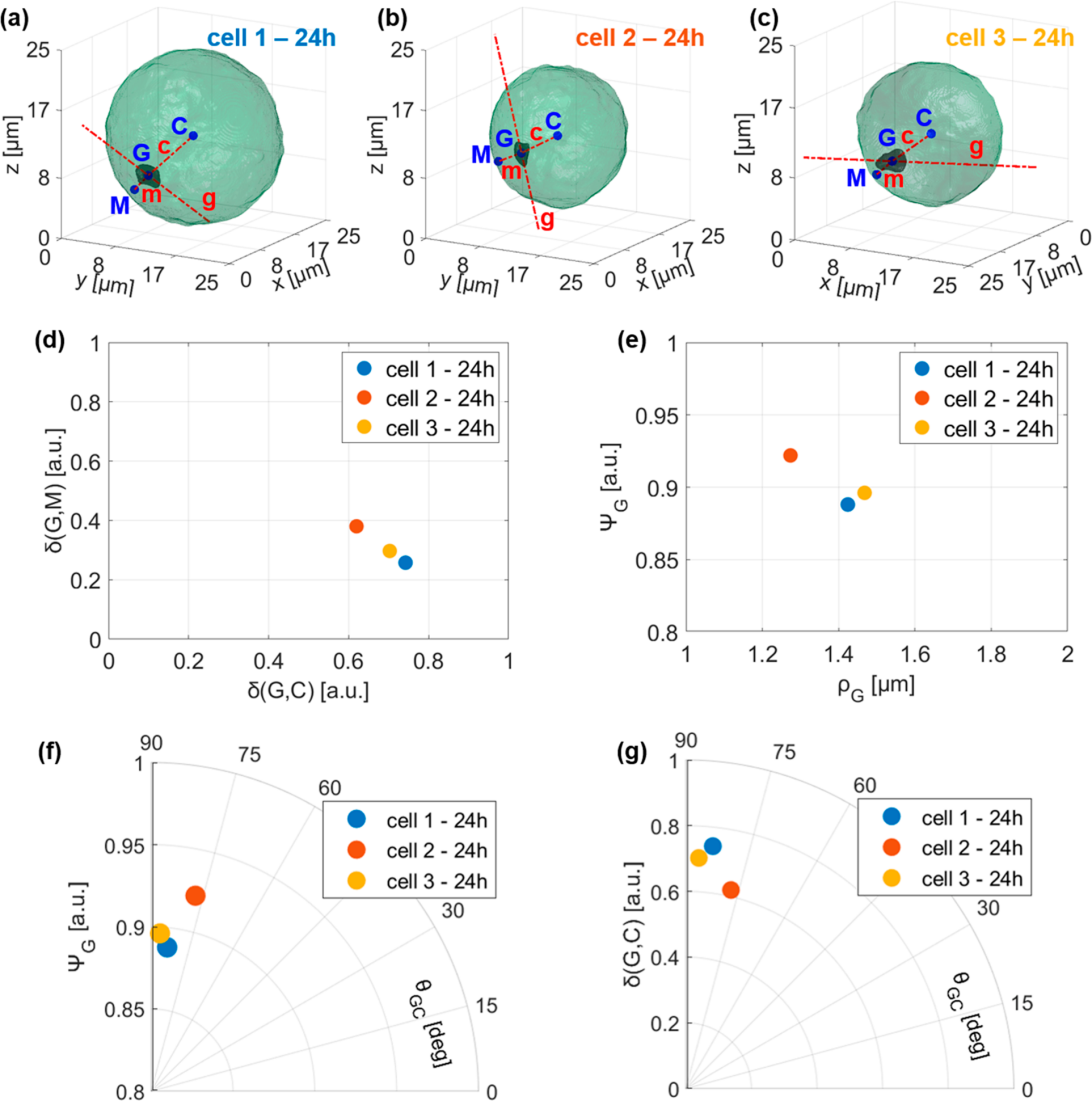
Three-dimensional quantitative Euclidean analysis from AM-TFC reconstructions of nGO uptake in NIH-3T3 cells after 24 h treatments. (a–c) AM-TFC reconstructions of three 24 h cells (green) and their nGO clusters (black). *C* is the cell centroid, *G* is the graphene centroid, and *M* is the nearest point of the external cell membrane to point G. Line *c* joins points *C* and *G* and line *m* joins points *M* and *G*. Line *g* passes for point *G* and is orientated like 3D nGO cluster. (d) Cartesian plot of graphene–cell normalized distance δ(*G*,*C*) versus graphene–membrane normalized distance δ(*G*,*M*). (e) Cartesian plot of graphene sphericity Ψ_G_ versus graphene equivalent radius ρ_G_. (f) Polar plot in which the radial coordinate is the graphene sphericity Ψ_G_ and the angular coordinate is the graphene–cell angle θ_GC_. (g) Polar plot in which the radial coordinate is the graphene–cell normalized distance δ(*G*,*C*) and the angular coordinate is the graphene–cell angle θ_*GC*_.

Furthermore, the test case reported in Figure 3 needs a more sophisticated morphological analysis. We model the 3D shape of nGO in Figure 3e as a toroid having its same volume, as shown by the inset in Figure 5a. The toroid provides a rough estimation of the nuclear size, since the 3D nGO ring distributes around the nucleus without accessing it, because nGO particles are larger than the functional diameter of the nuclear pores.^31^ This information is very valuable in a label-free technique such as DH, in which the intracellular identification is still an open and challenging issue, since no dyes are used to make the nucleus visible and easily detachable from the surrounding cytoplasm. Moreover, the toroidal modeling can be exploited for an additional quantitative analysis about 3D nGO distribution by unrolling its shape through the conversion in spherical coordinates, that is, azimuthal angle, elevation angle, and radial distance, as reported in yellow in Figure 5a. We perform the same calculation on the nGO shape, reported in black in Figure 5a, enabling a quantitative evaluation about the surface irregularity through histograms of spherical coordinates in Figure 5b–d. A more detailed discussion about the geometrical analysis and visualization is provided in Supporting Information.

**Figure 5:**
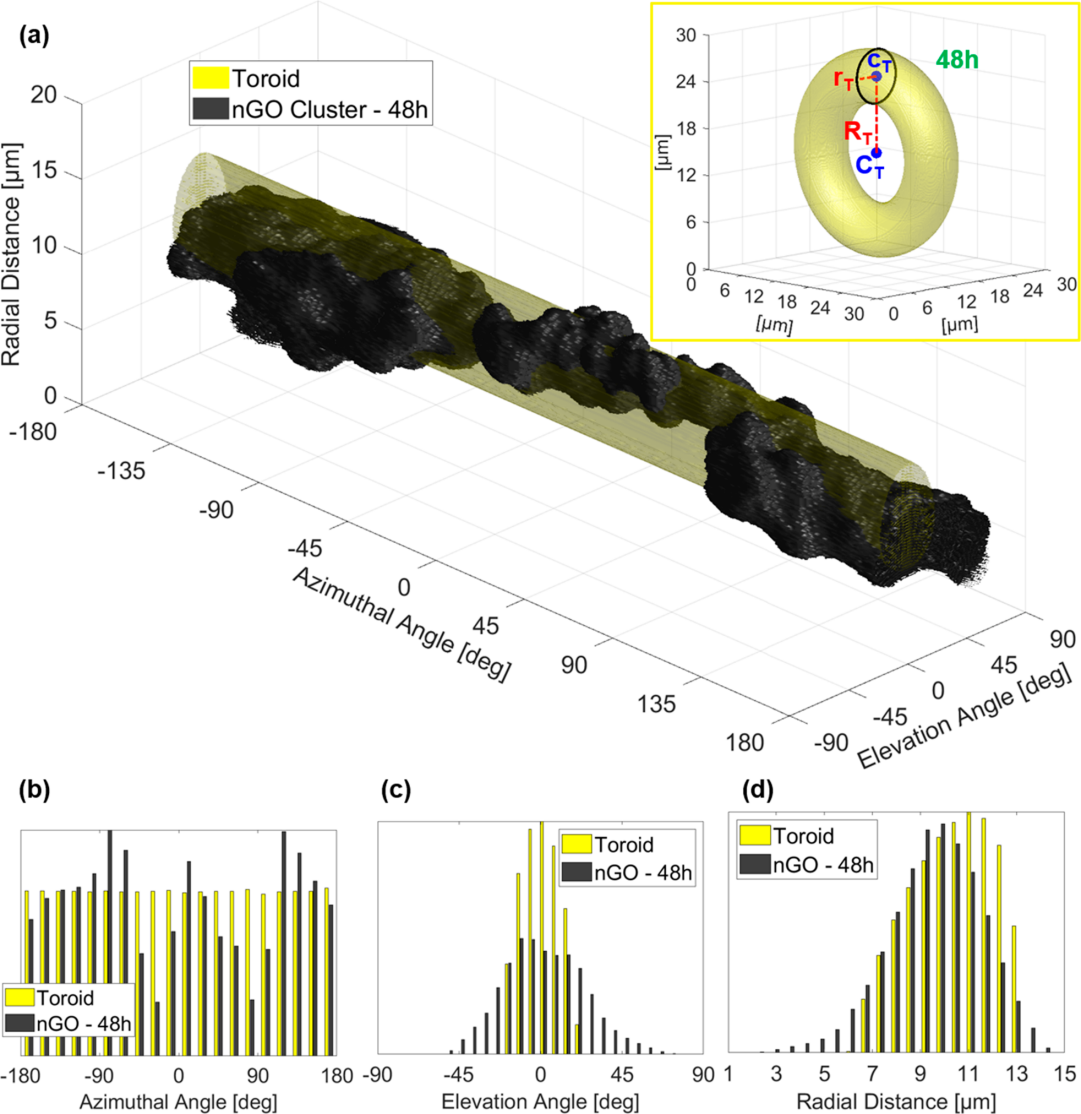
(See Supporting Movie 5) Three-dimensional quantitative Euclidean analysis from AM-TFC reconstructions of nGO uptake in NIH-3T3 cells after 48 h treatments. (a) Modeled toroid (yellow) and 3D nGO ring structure (black) unrolled by converting the Cartesian coordinates in spherical coordinates. In the inset, toroid used to model 3D nGO ring structure of the 48 h cell. *C*_T_ is the center of the toroid and *c*_T_ is the center of its generator circle (black), which radius is *r*_T_ (inner radius). The outer radius *R*_T_ is the distance between centers *C*_T_ and *c*_T_. (b–d) Comparison between the histograms of the modeled toroid (yellow) and the 3D nGO ring structure (black) about the azimuthal angle, the elevation angle, and the radial distance, respectively.

To summarize all of the quantitative descriptors, in Table 1 we report a set of parameters that can be quantitatively measured from the AM-TFC reconstructions at 24 and 48 h, including those described above. In particular, the graphene volume increases two orders of size in passing from 24 to 48 h. As a consequence, the cell equivalent radius grows by few microns, since a large amount of graphene is internalized. These volumetric measurements could be very useful to study the cytotoxicity effects due to nGO uptake, for example, to kill sick cells, such as tumor cells.^43^

**Table 1:** Quantitative Measurements in AM-TFC Reconstructions of Four NIH-3T3 Cells after 24 and 48 h from the nGO Adding in DMEM Medium

	24 h - 1	24 h - 2	24 h - 3	48 h
cell volume [μm^3^]	6006.59	3053.92	3092.40	9478.44
cell equivalent radius [μm]	11.28	9.00	9.04	13.13
graphene volume [μm^3^]	12.10	8.66	13.25	2339.69
graphene equivalent radius [μm]	1.42	1.27	1.47	8.24
graphene-cell volume ratio [%]	0.20	0.28	0.43	24.68
graphene surface area [μm^2^]	28.69	22.12	30.22	1933.38
graphene sphericity [%]	0.89	0.92	0.90	0.44
graphene first principal axis [μm]	3.47	2.85	3.60	
graphene second principal axis [μm]	2.90	2.47	2.64	
graphene third principal axis [μm]	1.72	1.77	2.08	
graphene-cell distance [μm]	8.35	5.51	6.25	1.79
graphene-membrane distance [μm]	2.90	3.38	2.64	
graphene-cell normalized distance [a.u.]	0.74	0.62	0.70	
graphene-membrane normalized distance [a.u.]	0.26	0.38	0.30	
graphene-cell angle [deg]	83.63	77.17	86.76	
toroid outer radius [μm]				9.88
toroid inner radius [μm]				3.47
azimuthal angle percentage error [%]				24.40
elevation angle percentage error [%]				54.82
radial distance percentage error [%]				30.72

The 3D spatial intracellular distribution of NPs clusters has been easily achieved for the first time by means of a new tomography method in flow microcytometry device. We demonstrated that the 3D spatial distribution of internalized nGO cluster can be easily quantified by retrieving the 3D tomogram of flowing cells, thus opening the way to high-throughput single-cell analysis. Reported proof-of-concept results show that a label-free, nondestructive, and fully automatic imaging technology is potentially able to provide statistically relevant assays on a large number of cells for better inference of new insights on how nGO becomes internalized and distributes itself inside them. Indeed, as cellular populations are heterogeneous and single-cell studies necessarily require nondestructive and label-free characterization methods, only a flow cytometry approach can allow thorough information on interaction of nGO at the intracellular scale. Moreover, the method we proposed can be extended without loss of generality to different classes of NPs and other cells. The AM-TFC concept has been introduced to cope with the issue of correctly visualizing 3D internalized particles, thus overcoming the limitations of PC-TFC due to absorption-related artifacts. Furthermore, we have shown that AM-TFC allows for better results in terms of resolution with respect to a well-established volume rendering technique, that is, SFS. Finally, we proposed a new set of geometrical descriptors from AM-TFC reconstructions to show that the proposed reconstruction approach permits measuring 3D morphometric features that otherwise cannot be accessed through any other existing label-free technique. They could be the basis for performing a statistical analysis of quantitative 3D biomarkers in order to measure and classify NP intracellular spatial distributions, thus exploring this new paradigm of 3D imaging at single-cell level, and also through high-throughput modality.

## Supporting Information Available

Additional details on cell culture (with Figure S1), tomographic flow cytometry by digital holography, shape from silhouette method, 3D morphological inspection of nGO within cells (PDF)
Movie of recorded DH sequence containing flowing and rotating NIH-3T3 cells (MP4)
Movie of recorded DH sequence, numerically retrieved QPMs and AMs, slice by slice and isolevels representation of both PC-TFC and AM-TFC reconstructions with highlighted in black the 3D nGO cluster localized in an NIH-3T3 cell after a 24 h treatment (MP4)
Movie of recorded DH sequence, numerically retrieved QPMs and AMs, slice by slice and isolevels representation of both PC-TFC and AM-TFC reconstructions with highlighted in black the 3D nGO cluster localized in an NIH-3T3 cell after a 24 h treatment (MP4)
Movie of recorded DH sequence, numerically retrieved QPMs and AMs, slice by slice and isolevels representation of AM-TFC reconstruction with highlighted in black the 3D nGO cluster localized in an NIH-3T3 cell after a 24 h treatment, while it is not identifiable in PC-TFC reconstruction (MP4)
Movie of recorded DH sequence after a 48 h treatment, numerically retrieved AMs, slice by slice and isolevels representations of AM-TFC reconstruction. The last part of the movie reports slice by slice and isolevels representations of the unrolled 3D nGO ring structure. The 3D nGO cluster localized in an NIH-3T3 cell is highlighted in black (MP4)
