# Supplementary material for: Three-Dimensional Quantitative Intracellular Visualization of Graphene Oxide Nanoparticles by Tomographic Flow Cytometry
Source: Nano Lett. 2021 Jul 7;21(14):5958–66. doi: 10.1021/acs.nanolett.1c00868 (PMC9297328; doi:10.1021/acs.nanolett.1c00868)
Supplement: Supplementary file 1 — nl1c00868_si_001.pdf [file nl1c00868_si_001.pdf]

Supporting Information for

3D Quantitative Intracellular Visualization of Graphene Oxide Nanoparticles by Tomographic Flow Cytometry

Daniele Pirone,[1,2,‡] Martina Mugnano,[1,‡] Pasquale Memmolo[1,] Francesco Merola,[1] Giuseppe Cesare Lama,[3] Rachele Castaldo,[3] Lisa Miccio,[1] Vittorio Bianco,[1] Simonetta Grilli,[1] and Pietro Ferraro[1,**]*

[1] CNR-ISASI, Institute of Applied Sciences and Intelligent Systems "E. Caianiello", Via Campi Flegrei 34, 80078 Pozzuoli, Napoli, Italy.

[2] Department of Electrical Engineering and Information Technologies (DIETI), University of Naples "Federico II", via Claudio 21, 80125 Napoli, Italy.

[3] CNR-IPCB, Institute of Polymers, Composites and Biomaterials, Via Campi Flegrei 34, 80078 Pozzuoli, Napoli, Italy.

[*] pasquale.memmolo@isasi.cnr.it , [**] pietro.ferraro@cnr.it

Cell culture

Murine embryonic fibroblasts NIH-3T3 were chosen to analyse the effects of nGO *in-vitro.* NIH-3T3 were grown in DMEM supplemented with 10% FBS (both Life Technologies, Carlsbad, CA), 2 mM L-glutamine (Sigma, St. Louis, MO), 100 U/mL penicillin, and 100 μg/mL streptomycin. Then, the fibroblasts were seeded at a cell density of 5×10^4 cells/mL in a 35 mm Petri dish (WillCo) and incubated at 37 °C and in a humidified 5% CO_2 atmosphere in an incubator (Esco). To investigate the 3D intracellular distribution of nGO, 50 μg/mL of nGO at intermediate oxidation degree (nGO2[31]) was added in the complete DMEM medium. Then, the cell culture was monitored at different time points at 24 h and 48 h, as shown by the inverted microscope images in Figs. S1(a,b), respectively. At time points of 24 h and 48 h, cells were detached by trypsin-EDTA and injected into a microfluidic channel to collect holographic images of flowing cells.

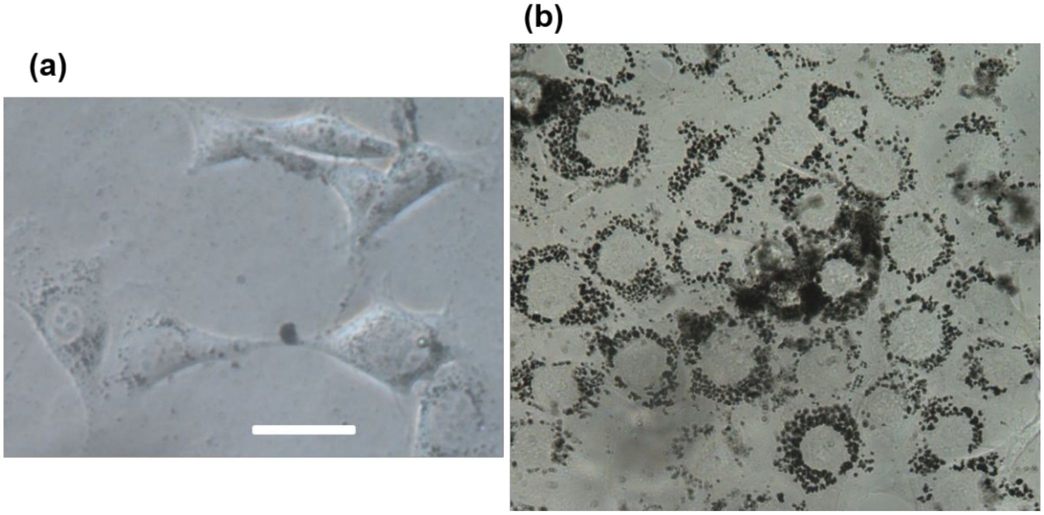

Figure S1. 2D imaging of NIH-3T3 cells by inverted microscope. (a,b) NIH-3T3 cells after 24 h and 48 h from the nGO adding in DMEM medium, respectively. The internalized nGO (black) increases with time and distributes around nucleus. Scale bar is 40 μm.

Tomographic flow cytometry by DH

A Mach-Zehnder interferometer in off-axis configuration is employed for the cell imaging. A beam-splitter divides the initial beam generated by a fiber-coupled laser (400 mW – 532 nm) into two beams, i.e., an object beam and a reference beam. A microfluidic pump pushes the biological sample into a microfluidic channel (PMMA polymeric material – length 7 cm – cross section 200 μm × 200 μm), generating a laminar flow at 12 nL/s. The corresponding parabolic velocity profile causes a torque on a cell flowing not at the channel's centre, thus inducing its rotation[S1]. After being collected by a microscope objective (Plan Apochromat – oil immersion 40× – numerical aperture 1.30), the object beam reaches the beam-combiner, thus interfering with the reference beam. The digital hologram corresponds to the resulting interference fringe pattern, that propagates up to a CCD camera (USB 3.0 u eye from IDS – 2048 × 2048 – pixel size 5.5 μm). Thanks to this configuration of the opto-fluidic setup, each cell has a full rotation inside a field of view of $0.042\ mm^2$. Potentially our recording system is able to inspect up to 100 cells/minute and, for each of them, over 100 holographic images at different viewing angles can be recorded. The duration of each experiment is about 3 minutes and 30 frames per second are collected. System calibration by standard USAF target is operated before each experiment in order to measure magnification, reconstruction pixel dimension, dependence of the magnification over the reconstruction distance, magnification homogeneity across the field of view, and axial resolution. Also beams alignment and fringe contrast are checked and optimized before experiments. In order to quantify the extent of noise on the acquired holograms, we measured the noise contrast over a homogeneous area of the amplitude of the demodulated hologram, i.e. where a flat background is in principle expected and any variation should be attributed to noise. The average measured noise contrast, defined as

the ratio between the standard deviation and the average value of the gray levels of the image, was C=0.18 in the case of the experiments reported in this paper.

Conventional optical tomography tools are based on two alternative sample probing strategies. In the first one[S2-S5], the sample (fixed) is scanned by an illumination beam which maximum viewing angle is however typically less than 150°. In the second one, the illumination beam direction is fixed, while sample can rotate thanks to mechanical[S6] or optical[S7] forces. The TFC principle belongs to this second type, but the imaged sample rotates due to the hydrodynamic effect within a microfluidic channel. Differently from conventional techniques, the orientation of cells is unknown, therefore suitable algorithms to estimate the rolling angles have been proposed. In particular, PC-TFC has been firstly demonstrated for the 3D RI inspection of red blood cells, exploiting the biolensing effect to calculate the rolling angles, and diatoms, for which the sample orientation was recovered by phase images similarities[33]. Recently, the PC-TFC of circulating tumor cells has been addressed by using the fluid modelling as the guide to calculate the 3D cell orientation[34,38]. In this paper, we employ the rolling angle recovery method proposed in ref. 38 but adapted to estimate the angles from amplitude images. In particular, the approach searches the frame corresponding to the image rotated of 180° with respect to the first one, by minimizing a similarity metric based on the Tamura coefficient[38]. Then, the remaining orientations are calculated as the incremental rolling angles with respect to the previous ones as a function of the cell movement along the flow direction. In summary, after the holographic video sequence recording of a flowing and rotating cell, the entire processing pipeline consists of

i) For each frame, numerical refocusing of the cell (position along the optical axis)[37].

ii) For each frame, transversal positioning of the cell from the in-focus AM reconstruction[37].

iii) 3D realignment in a fixed position of all AMs with respect their 3D coordinates calculated in i) and ii)[33].

iv) Estimation of the frame index f_{180} at which the 180° of rotation occurs with respect to the first frame of the sequence[38].

v) For the generic frame k, the rolling angle ϑ_k is calculated as $\vartheta_k = 180° \frac{y_k - y_1}{y_{f180} - y_1}$, where y_k is the k-th position along the flow direction[38].

vi) Given the AM images and the corresponding rolling angles, the tomographic reconstruction is performed by using the Filtered Back Projection algorithm[39], thus obtaining the AM-TFC images in Fig. 2 and Fig. 3.

All computational steps are performed using custom-made scripts in MATLAB R2020a on a desktop computer (Intel Core i7-4790 CPU, 3.6 GHz, 16 GB RAM). The reconstruction of each hologram of the recorded sequence employs about 5 sec. Therefore, the computational time to obtain a tomographic reconstruction depends on the number of the holographic projections (usually 50-100 images).

Shape From Silhouette method

The Shape From Silhouette (SFS) is a well-known and well-established method to recover the 3D shape of an object from their 2D projections taken at different viewing angles, proposed by A. Laurentini in ref. 35. Essentially, silhouettes are binary masks encoding the support (or foreground) of the imaged object from different directions, i.e. they are the regions where objects of interest project in images. Silhouettes extraction is a 2D-region image segmentation problem typically addressed by subtracting the background by using suitable thresholds. Given a single

silhouette image of an object, the 3D object lies inside the volume generated by back-projecting the silhouette area using the camera parameters[35]. With multiple views of the same object, the so-called generalized cones, generated by each image, can be intersected to build a volume which is guaranteed to contain the object. The smallest volume obtainable in this way is known as the visual hull of the object, that is the estimation of the 3D shape of the object. In our implementation of the SFS algorithm to obtain the result in Fig. 3(d), we set parallel silhouette cones, i.e. no effect of magnification is considered thanks to the 3D realignment in a fixed position of all the AMs with respect their 3D coordinates. Then, cone intersections are performed by using 68 viewing directions within the range [0, 180] degrees. In Figure S2, the sketch of the working principle of the SFS algorithm is report. More details about algorithms that can be used to calculate the visual hull can be found in ref. S8.

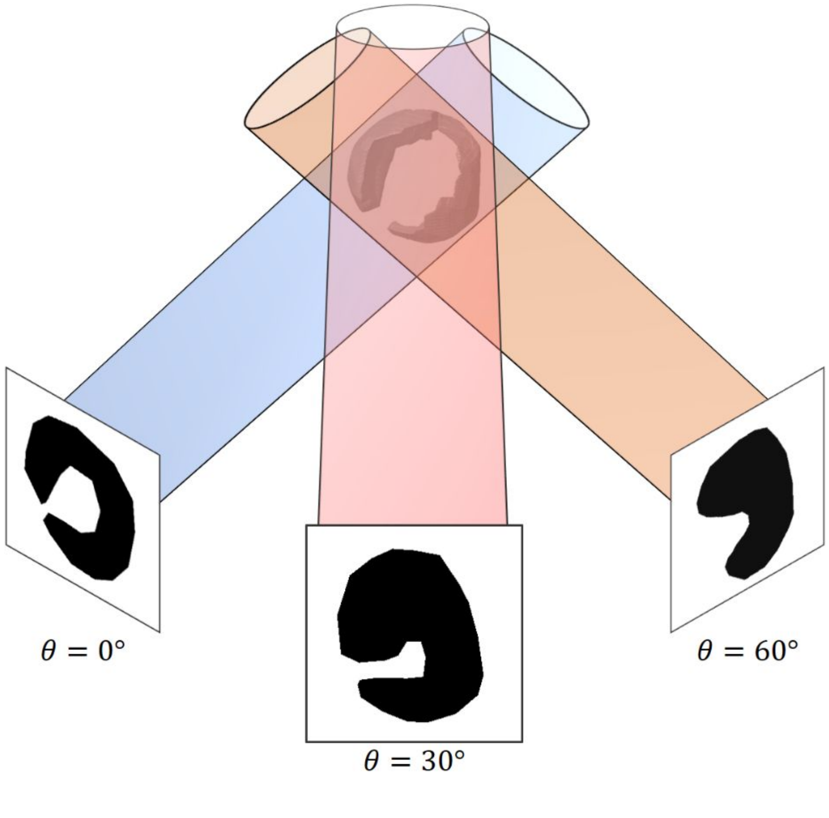

Figure S2. Sketch of the working principle of the SFS algorithm. For the sake of clearness, only three shape projections are depicted.

3D morphological inspection of nGO within cells

Let C be the cell centroid and G be the nGO cluster centroid. Let M be the nearest point of the external cell membrane to point G. The line passing for C and G is indicated by c, while the line passing for M and G is indicated by m. Moreover, let g be the line passing for point G and having as slope the 3D nGO cluster orientation. These geometrical elements are reported within AM-TFC reconstructions in Figs. 4(a-c) and they are used to define the following 3D morphometric features. We compute the graphene-cell normalized distance as

$$\delta(G,C) = \frac{\Delta(G,C)}{\Delta(G,C) + \Delta(G,M)}, \tag{S1}$$

and the graphene-membrane normalized distance as

$$\delta(G,M) = \frac{\Delta(G,M)}{\Delta(G,M) + \Delta(G,C)}, \tag{S2}$$

where $\Delta(\cdot,\cdot)$ is the Euclidean distance between two points. The normalized distances in Eq. S1 and Eq. S2 can take values from 0 to 1. In the three cells at 24 h, nGO clusters are about in the same relative position between cell centroid and cell membrane, as shown by the $\delta(G,C)$ versus $\delta(G,M)$ plot in Fig. 4(d). Moreover, nGO clusters appear much closer to cell membrane than cell center. This marginal position can be explained by considering that most of cell volume is occupied by nucleus, that in fact does not internalize nGO particles, because they are larger than the functional diameter of the nuclear pores[31]. Moreover, we use the sphericity Ψ_G as a synthetic descriptor parameter of the graphene shape, defined as[42]

$$\Psi_G = \frac{\sqrt[3]{\pi(6V_G)^2}}{A_G}, \tag{S3}$$

where V_G and A_G are the volume and the surface area of the nGO cluster, respectively. Therefore, Ψ_G measures how similar the shape of the nGO cluster is to that of a perfect sphere, and it takes

values in the range [0,1] (1 in the spherical case). In Figure 4(e), Ψ_G is reported versus the graphene equivalent radius ρ_G, i.e. the radius of a sphere with the same volume. On the bases of the defined descriptors, we infer that the nGO clusters in cell 1 and cell 3 have about the same equivalent radius and sphericity, while the nGO cluster in cell 2 is smaller with a quasi-spherical shape. Moreover, as displayed by the polar plot in Fig. 4(f), to the higher nGO sphericity Ψ_G corresponds a lower graphene-cell angle θ_{GC}, i.e. the angle between lines g and c in Figs. 4(a-c), which takes values from 0° to 90°. The lower graphene-cell angle θ_{GC} of cell 2 also corresponds to a lower graphene-cell normalized distance $\delta(G,M)$, as reported by the polar plot in Fig. 4(g). From Figures 4(a-c,f,g), it is clear that nGO clusters arrange within all three 24h-cells with an angle between 75° and 90° with respect to line c. Instead, for the 3D nuclear decoration observed within the cell in Fig. 3, a toroid model is used (see inset in Fig. 5(a)). Its volume can be computed as

$$V_T = 2\pi^2 R_T r_T^2, \tag{S4}$$

where R_T is the toroid outer radius, i.e. the distance between the centre of the tube c_T and the centre of the toroid C_T, and r_T is the toroid inner radius, i.e. the radius of the generator circle of the tube. We set V_T equal to the volume of the nGO ring structure in Fig. 3(e), while R_T is calculated as the mean distance between its centroid and all voxels. Then, r_T can be obtained by inverting Eq. (S4). By this way, the r_T value measures an estimation of the thickness of the nGO cluster, while R_T provides a rough estimation of the nuclear size, since the 3D nGO structure spreads around nucleus without entering within it[31]. By converting the cartesian coordinates in spherical ones (i.e. azimuthal angle, elevation angle and radial distance), the toroid can be unrolled, thus obtaining a cylinder, as represented in yellow in Fig. 5(a). The same transformation is done for the nGO ring structure of Fig. 3(e), as reported in black in Fig. 5(a). Beyond the clear visual comparison, the unrolling procedure shown in Fig. 5(a) enables a quantitative evaluation about the surface

irregularity of the nGO structure. Indeed, in the case of an ideal toroid, the azimuthal angle takes values in $[-180°,180°]$, the elevation angle takes values in $\left[-\tan^{-1}\left({}^{r_T}/{}_{R_T}\right),\tan^{-1}\left({}^{r_T}/{}_{R_T}\right)\right]$, and the radial distance takes values in $[R_T - r_T, R_T + r_T]$, as confirmed by their yellow histograms in Figs. 5(b-d). Same histograms (in black) are obtained for the nGO cluster in Figs. 5(b-d). To quantify the nGO cluster irregularities with respect to the toroid used as reference, we calculate the percentage error between histograms, resulting in 24.40% for the azimuthal angle, 54.82% for the elevation angle and 30.72% for the radial distance.

Supporting references

(S1) Torino, S.; Iodice, M.; Rendina, I.; Coppola, G.; Schonbrun, E. A microfluidic approach for inducing cell rotation by means of hydrodynamic forces. *Sensors* **2016**, 16, 1326.

(S2) Choi, W.; Fang-Yen, C.; Badizadegan, K.; Oh, S.; Lue, N.; Dasari, R. R.; Feld, M. S. Tomographic phase microscopy. *Nat. Methods* **2007**, 4, 717–719.

(S3) Kim, K.; Kim, K. S.; Park, H.; Ye, J. C.; Park, Y. Real-time visualization of 3-D dynamic microscopic objects using optical diffraction tomography. *Opt. Express* **2013**, 21, 32269-32278.

(S4) Isikman, S. O.; Bishara, W.; Mavandadi, S.; Yu, F. W.; Feng, S.; Lau, R.; Ozcan, A. Lens-free optical tomographic microscope with a large imaging volume on a chip. *Proc. Natl. Acad. Sci. U.S.A.* **2011**, 108, 7296-7301.

(S5) Pégard, N. C.; Toth, M. L.; Driscoll, M.; Fleischer, J. W. Flow scanning optical tomography. *Lab Chip* **2014**, 14, 4447-4450.

(S6) Charrière, F.; Marian, A.; Montfort, F.; Kuehn, J.; Colomb, T. Cell refractive index tomography by digital holographic microscopy. *Opt. Lett.* **2006**, 31, 178-180.

(S7) Habaza, M.; Gilboa, B.; Roichman, Y.; Shaked, N. T. Tomographic phase microscopy with 180° rotation of live cells in suspension by holographic optical tweezers. *Opt. Lett.* **2015**, 40, 1881-1884.

(S8) Matusik, W.; Buehler, C.; Raskar, R.; Gortler, S.J.; McMillan. L. Image-based visual hulls. *Proceedings of the 27th annual conference on Computer graphics and interactive techniques* **2002**, 369-374.

Supporting Movie 1 - recorded DH sequence containing flowing and rotating NIH-3T3 cells (MP4).

Supporting Movie 2 - recorded DH sequence, numerically retrieved QPMs and AMs, slice by slice and isolevels representation of both PC-TFC and AM-TFC reconstructions with highlighted in black the 3D nGO cluster localized in an NIH-3T3 cell after a 24h-treatment (MP4).

Supporting Movie 3 - recorded DH sequence, numerically retrieved QPMs and AMs, slice by slice and isolevels representation of both PC-TFC and AM-TFC reconstructions with highlighted in black the 3D nGO cluster localized in an NIH-3T3 cell after a 24h-treatment (MP4).

Supporting Movie 4 - recorded DH sequence, numerically retrieved QPMs and AMs, slice by slice and isolevels representation of AM-TFC reconstruction with highlighted in black the 3D nGO cluster localized in an NIH-3T3 cell after a 24h-treatment, while it is not identifiable in PC-TFC reconstruction (MP4).

Supporting Movie 5 - recorded DH sequence after a 48h-treatment, numerically retrieved AMs, slice by slice and isolevels representations of AM-TFC reconstruction. The last part of the movie reports slice by slice and isolevels representations of the unrolled 3D nGO ring structure. The 3D nGO cluster localized in an NIH-3T3 cell is highlighted in black (MP4).
